# Muscle metabolic responses to a 5v5 football game in trained female football players

**DOI:** 10.1007/s00421-025-06028-1

**Published:** 2025-10-22

**Authors:** M. B. Randers, J. Panduro, G. Ermidis, J. F. Vigh-Larsen, F. Yousefian, K. Søgaard, P. Krustrup, M. Mohr

**Affiliations:** 1 Department of Sports Science and Clinical Biomechanics, Faculty of Health Sciences, SDU Sport and Health Sciences Cluster, University of Southern Denmark, Campusvej 55, 5230 Odense M, Denmark; 2 Department of Movement and Sports Sciences, Faculty of Medicine and Health Sciences, Ghent University, Ghent, Belgium; 3 Department of Sport Sciences, Research Centre in Sports Sciences, Health Sciences and Human Development (CIDESD), University of Beira Interior, Covilhã, Portugal; 4 Danish Institute for Advanced Study (DIAS), University of Southern Denmark, Odense, Denmark; 5 Sport and Health Sciences, University of Exeter, Exeter, UK; 6 Center of Health Science, Faculty of Health, University of the Faroe Islands, Torshavn, Faroe Islands

**Keywords:** Muscle lactate, Muscle metabolism, Glycogen, Women´s soccer, Small-sided games, Intermittent exercise

## Abstract

**Purpose:**

The study examined the acute muscle and systemic physiological responses to a 5v5 small-sided game (SSG) of football in trained female players.

**Methods:**

Ten trained female football players (age: 24.5 ± 1.9 years, height: 169 ± 5 cm, weight: 67.0 ± 8.0 kg, fat%: 24.8 ± 7.6%) completed four 12-min periods of play (P1-P4) with 4-min passive recovery. Muscle biopsies were obtained from m. vastus lateralis pre- and post-game, and analyzed for glycogen, creatine phosphate (CP), ATP, and lactate. Blood lactate was measured pre-, mid-, and post-game. Heart rate and movement patterns were recorded continuously using chest-worn sensors.

**Results:**

Muscle lactate nearly doubled (mean [95CI], 4.8 [2.5, 7.1] to 8.8 [6.5, 11.0] mmol/kg dw; *P* < 0.01), while blood lactate rose 57% mid-game and 80% post-game (*P* < 0.01). Muscle CP declined 22% (*P* < 0.01), muscle ATP remained unchanged, and muscle glycogen declined (*P* < 0.01) from 349 [299, 399] at baseline to 275 [225, 325] mmol/kg dw post-game. Average and peak heart rates reached 82 [78, 86] % and 93 [89, 97] %HR_max_, respectively, while players covered 3805 [3308, 4303] m in total, with greater distance in the first period compared to later periods (P2–P4, *P* < 0.05).

**Conclusion:**

These findings indicate that 5v5 SSG impose a notable muscle metabolic load in trained female players, taxing both aerobic and anaerobic energy systems despite a relatively low external load. SSG may be a training modality for improving sport-specific fitness in trained young female players, but intervention studies are required to confirm adaptations. Inference is limited by the small sample and absence of control for menstrual phase and pre-exercise nutrition.

## Introduction

Physiological response to various football formats has been characterized more extensively and intensively in men, whereas investigations in women are sparse. Indeed, a recent bibliometric study demonstrated a 1:6 women-to-men ratio in football publications, while the ratio was as high as 1:10 for studies on physiological demands (Kirkendall and Krustrup 2022). Addressing this imbalance in football research, I practically important to better understand women´s football and to optimize women-specific training prescriptions and potentially reduce injury risk, studies with a physiological focus are warranted.

Previous studies in competitive female players have indicated that anaerobic energy turnover in female football players may be lower than in their male counterparts during a full 11v11 game as muscle lactate reaches 14.3 and 9.8 mmol/kg dw in females after intense periods of first and second halves, respectively, whereas values in males reaches 15.9 and 16.9 mmol/kg d.w. (Krustrup et al. 2005, 2022, 2006; Nybo et al. 2013). However, when comparing physiological markers of glycolytic activity in a simulated football model, female players appear to display similar levels (Bendiksen et al. 2012) as the men (Bendiksen et al. 2013). In untrained men and women, after 4 weeks of recreational football training, the increase in muscle lactate and the decrease in muscle glycogen were larger in men than women after a 7v7 recreational football game (Randers et al. 2010). Few studies have also shown that markers of oxidative stress are elevated after 11v11 football match play and futsal (Andersson et al. 2010; Souglis et al. 2023) with differences in response between men and women (Souglis et al. 2018). These observations underpin the high metabolic demands of football activities. No studies have to our knowledge investigated the muscle metabolic responses to small-sided games (SSG) in well-trained female football players, despite a broad consensus that SSG represent an effective training modality across multiple sports and populations (Halouani et al. 2014; Sarmento et al. 2018).

In untrained men aged 20–43 years, markedly higher anaerobic energy turnover was observed after 1-h SSG football training compared to 1 h of running (Krustrup et al. 2010). Furthermore, time in the high heart rate zones was substantially higher in football than running. Also, in a recent study in healthy young men, it was shown that ~ 1 h of SSG caused a marked rise in muscle and blood lactate and a ~ 20% decline in muscle glycogen (Panduro et al. 2022a, b). Thus, SSG football training appears to be a training modality that induces high cardiovascular loading while simultaneously activating the glycolytic energy pathway. However, this is yet to be determined in female players. Findings from both in vitro (Ro and Kaminska 2024) and in vivo studies (Russ et al. 2005) indicate sex differences in muscle metabolism during exercise, which may have a genetic and evolutionary origin (Landen et al. 2021; Mauvais-Jarvis 2024).

This study therefore aims at describing the acute muscle and systemic physiological response to a 1-h 5v5 SSG football in trained female players. We hypothesize that both anaerobic and aerobic energy systems are substantially taxed leading to substantial decrease in muscle glycogen levels. Primary endpoints are muscle and blood lactate, muscle CP, and glycogen, while secondary endpoints are heart rate and game activity data.

## Methods

### Participants

Ten well-trained female football players [mean age (± SD): 24.5 ± 1.9 years, height: 169 ± 5 cm, weight: 67.0 ± 8.0 kg, muscle mass: 27.8 ± 2.3 kg, fat%: 24.8 ± 7.6%] were recruited for the study. Inclusion criteria included no history of severe injuries, chronic diseases, or daily prescribed medications. Given the invasive biopsy design, sample size followed feasibility and aligns with prior field-based biopsy studies (Krustrup et al. 2022, 2006; Panduro et al. 2022a, b). A sensitivity analysis indicates that with *n* = 10 paired observations (*α* = 0.05, power 0.80), the minimum detectable standardized mean difference is ~ 0.92; observed effects for glycogen and CP exceeded this threshold. All participants received comprehensive written and verbal information about the study procedures, potential risks, and their rights as participants before providing written informed consent. All ten participants completed the study without any adverse events. No information regarding or control for menstrual phase, oral contraceptive use, or hormonal status was collected. The study received approval from the Regional Ethics Committee of Southern Denmark (S-20180139). The sample size was based on feasibility for repeated muscle biopsies and aligned with previous studies using similar invasive methods in elite athletes.

### Experimental design

The participants completed a 5v5 indoor football game on a wooden floor pitch organized as four times 12-min periods (P1, P2, P3, and P4) interspersed with 4-min resting periods. Prior to the game, a 15-min standardized warm-up comprising a modification of the FIFA 11 + warm-up recommendations (Bizzini and Dvorak 2015) and exercises with and without the ball were completed. Each 12-min period consisted of free play, with players self-refereeing, which is a common training modality in indoor football. All participants rotated as goalkeepers, ensuring that equal time spent in this role. Goalkeepers were allowed to participate in the play, and their movements were not restricted. While goal keeping may lower locomotor load, equal exposure across players likely minimized bias. The SSG were conducted on an indoor court (40 × 20 m, providing 80 m^2^ per player). Muscle biopsies from m. vastus lateralis were taken at rest (baseline) and at the end of the game. Blood lactate samples were collected at rest, after the second 12-min period (i.e., midway through the game), and at the end of the game. Player movement and cardiovascular strain were recorded using Polar Team Pro (Polar Team Systems, Polar Electro Oy, Kempele, Finland) sensors fixed to the chest. Teams were assigned by simple draw. With *n* = 10 stratification by positional roles or fitness level was not feasible and may have introduced slight imbalance. Upon arrival at the indoor facility, participants’ height and body composition were assessed using a Leicester transportable stadiometer (Tanita, Denmark) and a bioimpedance scale (InBody270, InBody Danmark, Copenhagen, Denmark). Participants were instructed to arrive euhydrated and consume a light meal 2–3 h prior to arrival. Diet and hydration were not standardized or recorded.

### Muscle biopsies

Muscle biopsies were collected to analyze muscle glycogen, lactate, pH, adenosine triphosphate (ATP), and creatine phosphate (CP). Biopsies were collected from the m. vastus lateralis of the participants' nondominant leg, both pre (at rest) and post-SSG. Participants were instructed to avoid strenuous activity for at least 48 h before testing and to consume a light meal 2 h prior to the session.

Biopsies were taken with participants in a supine position in a room located approximately 15 m from the playing field. A small incision was made in the m. vastus lateralis under local anesthesia (1% lidocaine), and approximately 0.1 g of muscle tissue was extracted using a Bergström needle with suction (Bergström, 1962). At the end of the 1-h SSG, players were removed from the game one at a time for post-SSG biopsy sampling. Due to the time required for each biopsy, the first participant left the match 6 min into the fourth period, and afterward, the player returned to the game. The total time for all post-SSG biopsies was approximately 6 min. To maintain the 5v5 game format during each player’s absence, a staff member acted as a replacement. The match continued until all players had post-SSG biopsies taken. Due to the proximity of the biopsy room (15 m from the court), participants were instructed to jog from the pitch to the biopsy room at a moderate pace. The biopsy procedure took 15–30 s once the participant was lying down.

### Muscle sample analysis

Immediately after extraction, muscle biopsies were frozen in liquid nitrogen and stored at − 80 °C until analysis. Samples were freeze-dried for 48 h, and then carefully dissected to remove blood, fat, and connective tissue. The samples were weighed on a digital scale (Mettler AT261 DeltaRange®, Mettler Toledo, Leicester, United Kingdom) and divided into smaller portions for the analysis of glycogen and metabolites. For glycogen analysis, 1–2 mg of dry weight (d.w.) was hydrolyzed at 100 °C in 0.5 ml 1 M HCl for 2.5 h, followed by a 10-min centrifuge at 3500 g and 4 °C to remove undissolved material. The supernatant was stored at -80 °C until analysis via the hexokinase method (Passonneau and Lowry 2008) using a spectrophotometer (Beckman DU-650, Beckman Coulter, Life Sciences, Indianapolis, United States). For metabolite analysis, approximately 4 mg d.w. of muscle tissue was combined with 0.5 M PCA and 1 mM EDTA, mixed for 10 min, and then centrifuged at 13,600 g and 4 °C for 3 min. After neutralization to pH 7.0 with 2.1 M KHCO3 and further centrifugation, the supernatants were stored at − 80 °C for later analysis (Passonneau and Lowry 2008) via spectrophotometry (Beckman DU-650, Beckman Coulter).

### Blood lactate measurement

Blood lactate levels were measured at rest before the warm-up (Pre), midway through the game (immediately after the second 12-min period; mid), and immediately following the fourth period of SSG (Post). Samples were collected from a finger capillary and analyzed immediately using a Lactate Scout Monitor (EKF Diagnostics, Cardiff, Great Britain), with the sample obtained via sensor stick (Lactate Scout, SensLab GmbH, Leipzig, Germany).

### Heart rate monitoring and activity pattern tracking

Each participant wore a Polar Team Pro sensor (Polar Team Systems, Polar Electro Oy, Kempele, Finland) equipped with a heart rate monitor and integrated accelerometer. Heart rate was recorded every second using short-range radio telemetry, and movement was captured at a frequency of 200 Hz by the accelerometer. Participants wore the equipment from the beginning of the warm-up through the entire testing period. The Polar Team Pro device was positioned horizontally on a belt around the chest, centered below the sternum, in accordance with manufacturer guidelines. This device enabled continuous monitoring of players' movement patterns and overall body load in real time, with data collected on Polar software and exported to Microsoft Excel. Distance covered was categorized into three speed zones: low-intensity (< 9 km/h), moderate-intensity (9–13 km/h), and high-intensity running (> 13 km/h). Specific actions were evaluated using video analysis recorded by a digital camera (Sony-DCR-SX65E, Tokyo, Japan). Movement specific actions included passes, dribbles (take-ons), shots, tackles with the foot, and tackles with the upper body. These analyses were performed by an experienced researcher, who have analyzed more than 300 match observations. No separate inter- or intra-rater assessments were performed for this dataset.

### Statistics

All statistical analyses were conducted in RStudio (ver. 25.05.1). Linear mixed-effects models were used to evaluate changes over time in physiological, technical, and locomotor variables. Time (period or match phase) was modeled as a fixed effect, and subject ID was included as a random intercept. Depending on the variable, models included two (Pre/Post), three (Pre/Mid/Post), or four levels (P1–P4). Type III ANOVA tables were generated using the Satterthwaite approximation. Given *n* = 10, mixed-model estimates may be imprecise. Therefore, we report estimated marginal means (EMMs) with 95% CI except participant characteristics, which are presented as raw means ± SD. Pairwise contrasts were conducted using the Kenward–Roger approximation for degrees of freedom. Where appropriate, P values were adjusted using Bonferroni correction. Effect sizes were calculated as partial eta squared (ηp^2^) for ANOVA with repeated measures, whereas standardized effect sizes (Hedges’ *g*) were calculated from model residuals for pairwise comparisons. Where relevant, relative changes from baseline values were expressed as percentage differences and individual trajectories are displayed. A significance level of 0.05 was chosen.

## Results

### Muscle and blood metabolites

**Fig. 1:**
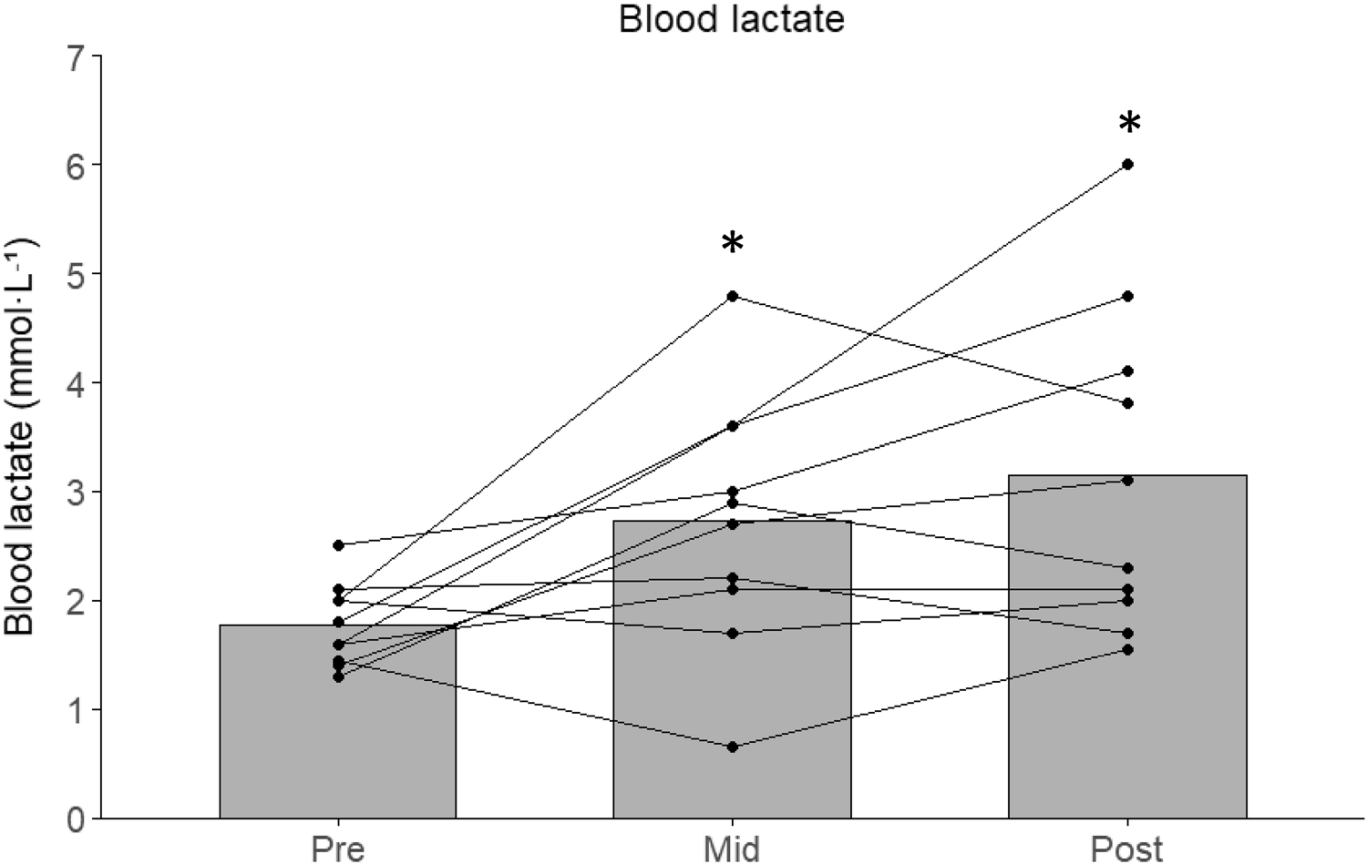
Blood lactate concentration before (Pre), midway through (Mid), and immediately after (Post) a 5v5 small-sided football game in trained women players (*n* = 10). Bars represent group means, and individual responses are shown by connected dots. * denotes a significant difference from Pre (*P* < 0.05)

**Fig. 2:**
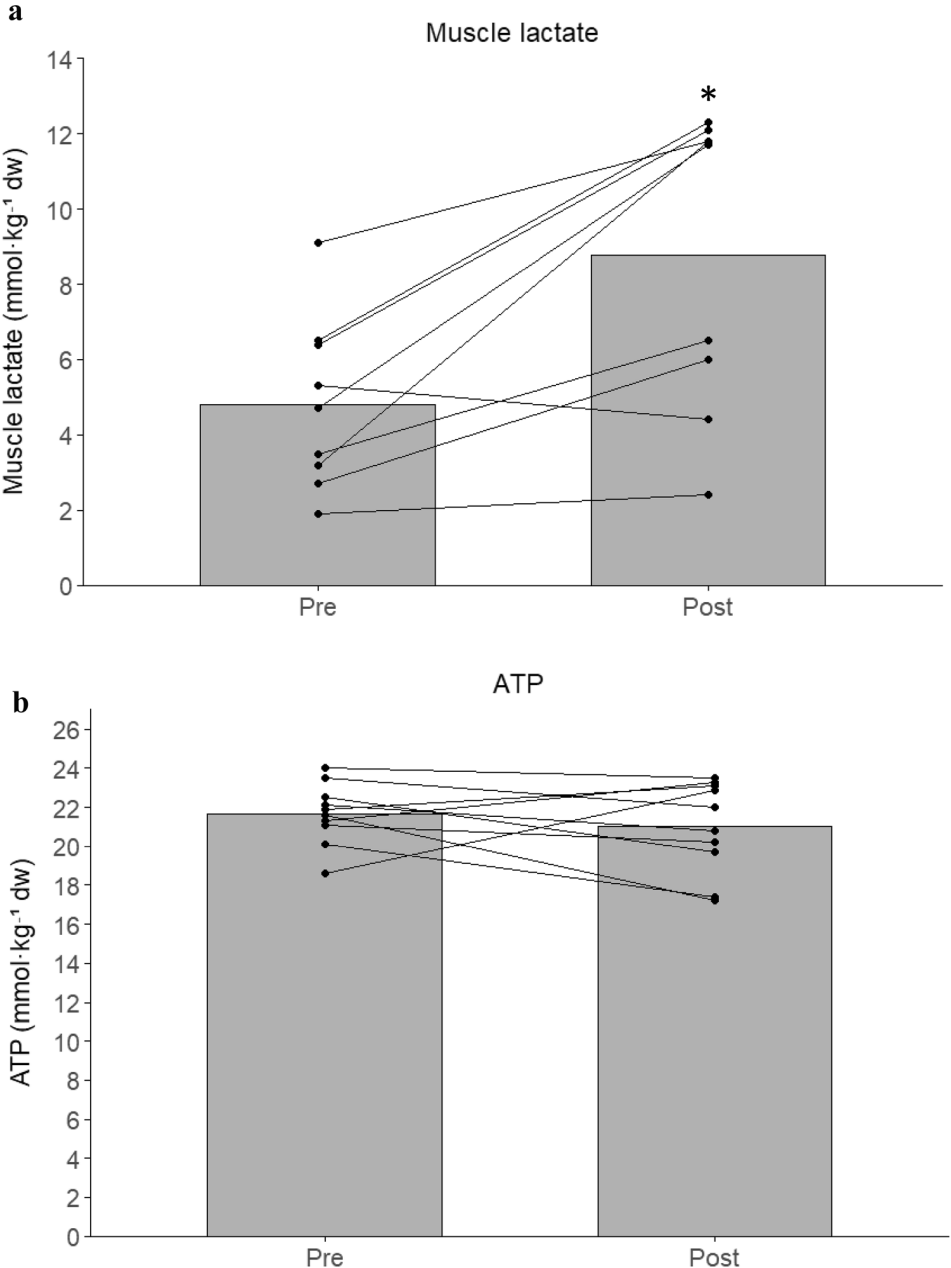

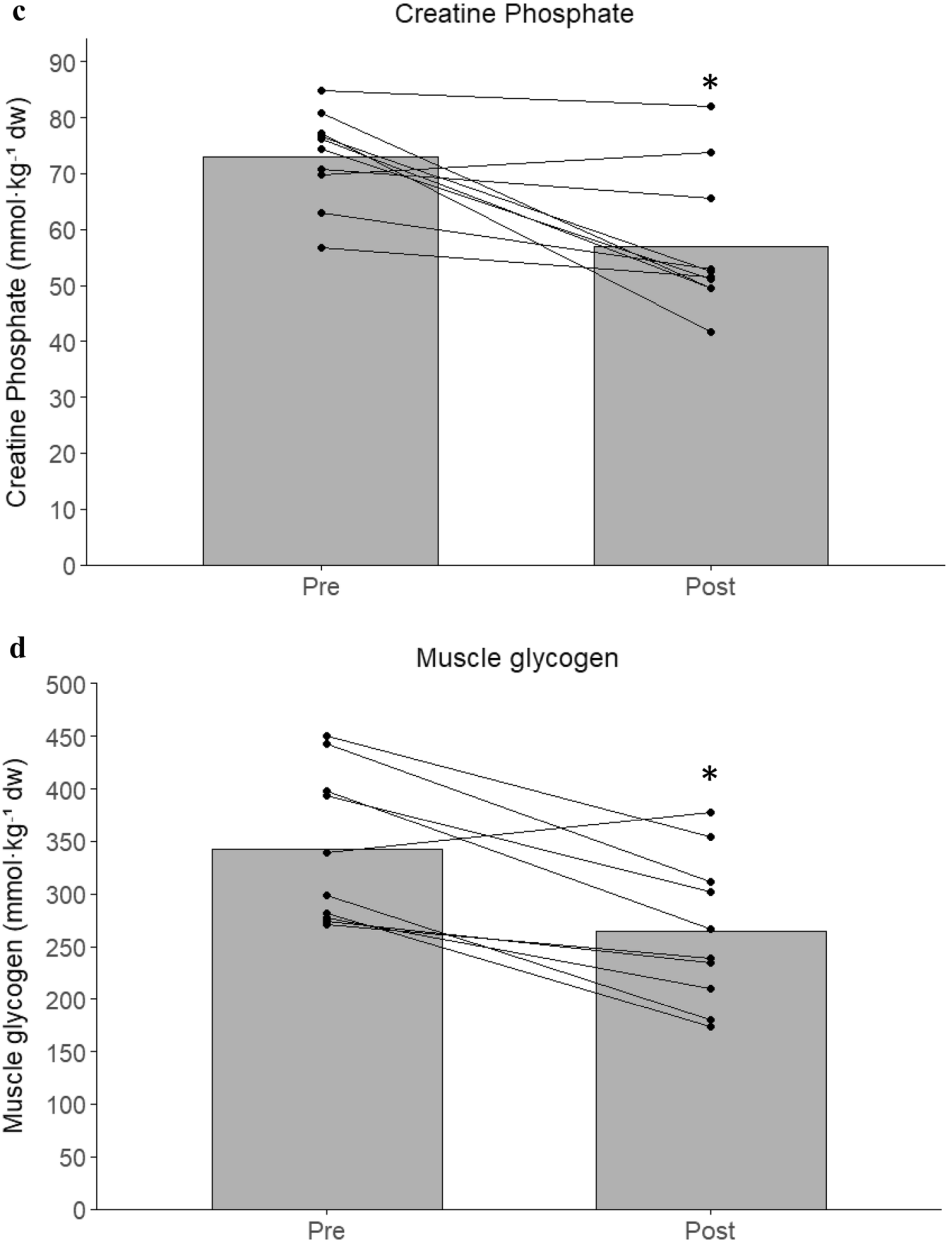
Muscle metabolite concentrations before (Pre) and immediately after (Post) a 5v5 small-sided football game in trained women players (*n* = 10). (**a**) Muscle lactate, (**b**) muscle ATP, (**c**) muscle creatine phosphate (CP), and (**d**) glycogen concentrations. Bars represent group means, and individual values are connected by lines. * denotes a significant difference from Pre (*P* < 0.05)

Muscle lactate nearly doubled from pre- to post-game (94 ± 83%; *P* < 0.01; *g* = 1.83; Fig. 2a). Blood lactate also increased significantly over time (ηp^2^ = 0.42) rising 57 ± 68 and 80 ± 84% at mid- and post-game (*P* < 0.01; Fig. 1). Muscle ATP remained unchanged (Fig. 2b). In contrast, muscle CP decreased by 22% (*P* < 0.01; *g* = 1.74; Fig. 2c), while muscle glycogen was 348 [299, 399] mmol·kg⁻^1^ dw at rest and decreased by 22 ± 15% post-game (*P* < 0.01; *g* = 1.88; Fig. 2d).

### Heart rate

Average and peak heart rates during the game were 166 [157, 175] bpm and 189 [175, 193] bpm, corresponding to 82 [78, 86]% and 93 [89, 97]% of HR_max_, respectively. Time spent in heart rate zones 80–90 and > 90% of HR_max_ was 44 ± 18, and 21 ± 24% of the playing time, respectively. Mean heart rate decreased (*P* < 0.001; ηp^2^ = 0.48) across the four 12-min periods (85[80, 90], 81[76, 86], 80[75, 85], 80[75, 85] %HR_max_, P1–P4, respectively), with values higher in P1 than all the three following periods (*P* < 0.05; *g* = 1.48–2.20).

### Activity profile and technical actions

Total distance covered during the game was 3805 [3308, 4303] m equal to 79.3 [68.9, 89.6] m/min with 3066 [2655, 3477] at low speed, 546 [449, 643] at moderate speed, and 194 [116, 271] m at high-speed equaling 63.9 [55.3, 72.4], 11.4 [9.4, 13.4], and 4.0 [2.4, 5.6] m/min, respectively. Peak speed reached 22.9 [20.9, 24.8] km/h, while peak acceleration and deceleration were 2.51 [2.12, 2.90] and − 1.73 [− 1.85, − 1.62] m/s^2^, respectively.

The total number of passes, dribbles, and shots across the match were 48 [44, 53], 8 [4, 12], and 7 [5, 9], respectively. Tackles with the upper body and the foot totaled 2 [1, 3] and 5 [3, 7], respectively. Period-specific locomotor and technical data are presented in Table 1.

**Table 1:** Physical and technical performance variables across four periods (P1–P4) of a 5v5 small-sided football game in trained women players (*n* = 10)

	**1st period (P1)**		**2nd period (P2)**		**3rd period (P3)**		**4th period (P4)**		**Time effect**	**Effect size**
	EMM	95% CI	EMM	95% CI	EMM	95% CI	EMM	95% CI	*P*	ηp^2^
Total distance (m)	1017	[893, 1140]^b,c^	920	[796, 1043]^a^	924	[801, 1048]^a^	945	[821, 1068]	**0.007**	0.357
Low-speed distance (m)	796	[691, 900]	771	[666, 876]	748	[643, 852]	752	[647, 857]	0.426	0.096
Moderate-speed distance (m)	175	[147, 202]^b,c,d^	119	[92, 147]^a^	127	[99, 154]^a^	125	[98, 153]^a^	** < 0.001**	0.480
High-speed distance (m)	46	[24, 69]	29	[7, 52]^d^	50	[27, 73]	67	[45, 90]^b^	**0.016**	0.311
Peak acceleration (m s^−2^)	2.16	[1.87, 2.44]	2.12	[1.84, 2.41]	2.06	[1.77, 2.34]	2.14	[1.86, 2.43]	0.951	0.009
Peak deceleration (m s^−2^)	− 1.91	[− 2.13, − 1.70]	− 2.06	[− 2.27, − 1.84]	− 1.86	[− 2.07, − 1.64]^d^	− 2.21	[− 2.43, − 2.00]^c^	**0.024**	0.291
Passes (n)	12.7	[11.0, 14.4]	12.5	[10.8, 14.2]	11.5	[9.8, 13.2]	12.0	[10.3, 13.7]	0.673	0.049
Dribbles (n)	2.9	[1.9, 3.9]^b^	1.4	[0.4, 2.4]^a^	1.9	[0.9, 2.9]	1.8	[0.8, 2.8]	**0.016**	0.287
Shots (n)	2.2	[1.4, 3.0]	1.5	[0.7, 2.3]	2.0	[1.2, 2.8]	1.3	[0.5, 2.1]	0.126	0.171
Tackles with the upper body (n)	0.4	[0.0, 0.8]	0.4	[0.0, 0.8]	0.6	[0.2, 1.0]	0.4	[0.0, 0.8]	0.848	0.026
Tackles with the foot (n)	1.2	[0.5, 1.9]	1.4	[0.7, 2.1]	1.5	[0.8, 2.2]	0.8	[0.1, 1.5]	0.377	0.097

Data are presented as estimated marginal means and 95% confidence intervals. Main effect for time reflects repeated-measures ANOVA across periods

Effect size is presented as partial eta squared (ηp^2^)

Superscripts denote significant (*P* < 0.05) pairwise differences: a = significantly different from 1st period (P1), b = from 2nd period (P2), c = from 3rd period (P3), d = from 4th period (P4)

Low-speed running (< 9 km/h), moderate-speed running (9–13 km/h), and high-speed running (> 13 km/h)

## Discussion

The present study provides novel insights into the physiological demands of 5v5 SSG in trained female football players. Our primary endpoints show that the anaerobic energy pathways are activated, and muscle glycogen moderately (− 22%) degraded. According to the secondary endpoints, the aerobic loading is also moderate to high as evidenced by average and peak heart rates of 82 ± 7% and 93 ± 4% of HRmax. While average and peak HR values suggest a substantial cardiovascular demand, the wide variability in time spent > 90% HRmax (21 ± 24%) indicates large inter-individual differences in aerobic strain, a normal finding in team sport research. Similar inter-individual variability has also been observed in biochemical responses, where elite female players showed marked differences in muscle damage and oxidative stress markers following a competitive match (Gravina et al. 2011), underlining that metabolic strain can vary considerably between players. The general work rate appears to be reduced after the first 12-min period, primarily due to reduced moderate-speed running, while high-speed running remained stable or even increased late in the session. These findings indicate that SSG training imposes meaningful metabolic and cardiovascular strain on trained female players, taxing both aerobic and anaerobic pathways—including glycolytic and alactic systems—contributing to reductions in muscle glycogen and CP, which may compromise work rate.

Markers of glycolytic activity indicate moderate activation of the anaerobic glycolytic pathway, as reflected by the rise in muscle lactate (~ 5 to ~ 9 mmol per kg dw). This is lower than observed in elite female players after a 11v11 game, where muscle lactate was ~ 13 mmol per kg dw after the game, with even higher values measured after intense periods in the game (Krustrup et al. 2022). In accordance, the blood lactate concentrations (~ 3 mmol/L) were also markedly lower in the present study than observed by Krustrup et al. (2022) during a full-sized game reaching values two-to-threefold higher. First, these differences may be explained by the very low distance covered with high-speed running (> 13 km/h) in the 12-min periods prior to measurements. Indeed, only 29 m and 67 m were covered with speed above 13 km/h during the second and fourth 12-min periods, whereas distances of 150–400 m were covered above 13 km/h in the last 15 min of the game prior to measurements in the study by Krustrup et al. (2022) (see also Mohr et al. (2022)). Similar distances have been observed during the last 15 min of official matches in the top Danish women’s league (Panduro et al. 2022a, b). Thus, despite considerable inter-match variability in external load metrics (Baptista et al. 2022), high-intensity distances are markedly higher during 11v11 matches than those observed during the present 5v5 SSG. Second, since the capacity of the glycolytic system is highly affected by training status (Hostrup and Bangsbo 2023), and the players in the studies by Krustrup et al. (2022) and Mohr et al. (2022) were elite players from the top tier in Greece with VO_2max_ > 50 ml kg^−1^ min^−1^ and a Yo-Yo Intermittent Recovery level 1 test score of ~ 1100 m, it is likely that their physiological capacity was markedly higher than that of the participants in the present study even if this was not measured in the current study.

Muscle glycogen depletion, which is highly linked to high-intensity performance (Mohr et al. 2022; Vigh-Larsen et al. 2022, 2021, 2025), was 78 mmol/kg dw during the game corresponding to a 22% drop after 48 min of 5v5, which is markedly lower than after 96 min of 11v11, in which muscle glycogen dropped by 173 mmol/kg dw equaling a 42% reduction (Krustrup et al. 2022). However, when adjusting for exercise time, the muscle glycogen degradation rate was only ~ 10% higher in the 11v11 game. It should be noted that these calculations do not account for potential glycogen resynthesis during lower intensity exercise periods or the three rest intervals stimulated by the elevated lactate concentrations (Nordheim and Vøllestad 1990). Thus, taken together, even though distance covered with high speed was limited, the anaerobic energy system was taxed to some extent during the 5v5 game format resulting in moderate muscle glycogen utilization. The high number of brief intense specific actions, such as passes, shots, dribbles (take-ons), as well as near maximal brief accelerations and decelerations, is likely to contribute significantly to the anaerobic energy turnover and breakdown of muscle glycogen (Vigh-Larsen et al. 2021; Vigh‐Larsen et al. 2025).

Women may exhibit slightly different responses due to sex-based physiological differences, such as lower muscle mass, fiber-type distribution, and hormonal variations influencing metabolism (Besson et al. 2022; Hunter and Senefeld 2024), which most likely has a natural selection origin (Mauvais-Jarvis 2024). These physiological characteristics may also influence energy system utilization and substrate turnover during high-intensity exercise. Esbjörnsson-Liljedahl et al. (1999) demonstrated that women exhibit significantly less glycogen degradation and lactate accumulation in type I fibers during sprint exercise, despite similar ATP and CP depletion in both fiber types. This suggests that differences in glycolytic activation may be more pronounced in oxidative fibers and could contribute to the moderate lactate response observed in the present study despite high internal load. These fiber-type differences may also influence training adaptation, fatigue resistance, and metabolic flexibility during repeated high-intensity efforts (Ansdell et al. 2020), such as those in SSG. Studies have shown that women generally rely more on lipid oxidation and may experience slower glycogen depletion rates (Holcomb et al. 2022; Tarnopolsky 2008), yet our findings reveal that trained female players also experience significant glycogen degradation. A comparable reduction in muscle glycogen (72 mmol/kg dw, corresponding to 21%) was observed after a similar organized 5v5 game for men (Panduro et al. 2022a, b). Muscle and blood lactate at the end of the game were also only slightly higher (9.6 vs. 8.8 mmol/kg dw and 3.8 vs. 3.1 mmol/l, respectively) in the men. It is notable that the degree of muscle glycogen depletion and lactate accumulation were comparable between men and women, as the men completed 32% longer total distance and 5.5-fold the distance covered with high-speed running (> 13 km/h) (Panduro et al. 2022a, b). The huge difference observed in high-speed running may partly be explained by differences in maximal running speed, placing the threshold at a lower relative speed for men compared to women. Similarly, men generally possess a higher aerobic capacity and higher anaerobic thresholds (Hunter and Senefeld 2024), therefore enabling them to sustain a higher running speed while taxing the anaerobic system to a similar extent. Some studies have indicated lower anaerobic energy turnover and higher reliance on the aerobic energy system in women than men during a full 11v11 game (Krustrup et al. 2005, 2022, 2006), whereas women show comparable levels of physiological markers of glycolytic activity during a simulated football model (Bendiksen et al. 2012, 2013). In trained male football players, beta-hydroxyacylCoA-dehydrogenase (HAD) protein expression showed a strong correlation to high-intensity running in the final 15-min period of a game (Mohr et al. 2016), whereas HAD did not correlate with any game performance parameters in women (Mohr et al. 2022). It was therefore speculated that the glycogen sparing effect of a high fat oxidation capacity may be less important in women’s football. However, the responses observed in the current study, which were similar to the male players in Panduro et al. (2022a, b), support the studies showing only minor differences between sexes in the physiological response to SSG.

In the current study, muscle CP was lowered by 22% (to ~ 55 mmol·kg^−1^ d.w.) in the female players. It should be mentioned that due to a ~ 30 s delay in the muscle sampling. Based on previously reported muscle CP resynthesis rate of 0.5 mmol·kg^−1^ d.w.s^−1^ after intense exercise (Bogdanis et al. 1998, 1995), it could be speculated that the actual concentrations are likely to be around 40 mmol·kg^−1^ d.w, similar to levels reached after a 20-m sprint test (Bogdanis et al. 1998; Fiorenza et al. 2019). Thus, the high number of short explosive movements during SSG is likely to cause high activations of the creative kinase reaction, which may also help explain the concurrently moderate-to-high aerobic demands observed, as oxidative phosphorylation supports CP resynthesis between bouts (Bogdanis et al. 1995). Indeed, the aerobic loading during 5v5 SSG was similar to what has been observed in men (HRmean: 166 vs 169 bpm, and HRpeak: 189 vs 190 bpm in women and men, respectively), and confirms a vast amount of research stating that SSG are highly demanding for the aerobic energy system (Beato et al. 2025; Halouani et al. 2014; Sarmento et al. 2018).

A notable finding is the apparent dissociation between internal and external load, with high heart rates and moderate muscle glycogen and muscle CP depletion occurring despite relatively low total and high-speed running distances (~ 3.8 km and < 200 m, respectively). This mismatch likely reflects the high frequency of short-duration, high-intensity actions—such as accelerations, decelerations, and technical efforts—that are metabolically taxing despite limited body displacement. These findings are consistent with the previous observations in SSG formats, where physiological strain is driven more by movement frequency and neuromuscular demands than by traditional distance metrics, especially on smaller pitches (Randers et al. 2020, 2018).

Considering the observed distance covered with high-speed running across the four periods, with higher distance in the fourth than the second period, it is evident that the relationship between glycogen depletion and high-intensity activity is complex. While overall work rate was highest during the first period, primarily caused by significantly higher moderate-speed running distance, the high-speed running distance showed variability rather than a straightforward decline. This pattern suggests that although muscle glycogen depletion likely contributes to reduced work rate, players can maintain the ability to perform brief bursts of high-speed effort in later periods. This aligns with other studies showing that athletes may preserve capacity for intermittent high-intensity activities even as overall endurance diminishes (Mohr et al. 2012), though additional stressors, such as oxidative stress and muscle damage, may further compromise performance and recovery (Andersson et al. 2010; Souglis et al. 2018, 2023).

The study has some strengths and some limitations. From a practical standpoint, understanding metabolic responses in SSG is important for avoiding overreaching, especially in congested calendars or high training loads (Meeusen et al. 2013). Monitoring such physiological strain may inform more sex-specific approaches to load management (Ansdell et al. 2020). A unique strength of the present study is the use of muscle biopsies to directly quantify glycogen, creatine phosphate, ATP, and lactate responses during SSG in trained female players. Such invasive data are rarely available in women’s football and provide novel insight into internal load that cannot be captured by external metrics alone. The parallel inclusion of heart rate and locomotor data further strengthens the interpretation of internal and external load relationships. Key limitations include whole-muscle rather than fiber-type-specific analysis of muscle glycogen (Vigh-Larsen et al. 2022, 2021). Moreover, we did not collect information regarding or controlled for menstrual phase, oral contraceptive use, or hormonal status. Moreover, pre-exercise diet was not controlled, although players were urged to eat a light meal 2–3 h prior to game. Another limitation of this study is the ~ 6-min window for post-game muscle biopsies, which introduced slight inconsistencies in timing of post-sampling. This difference in biopsy timing and the time delay may have led to partial resynthesis of time-sensitive metabolites, such as CP and lactate, potentially introducing further inter-individual variance due to differences in game demands prior to the biopsy. A priori power analysis was not performed due to the exploratory nature and logistical constraints of invasive testing. While the sample size (*n* = 10) limits generalizability and increases the risk of Type II error, the effect sizes for key outcomes (e.g., glycogen and CP) were large, supporting the physiological relevance of the findings.

In conclusion, this study indicates that 5v5 SSG impose moderate-to-high metabolic and cardiovascular demands in trained female football players, taxing both the anaerobic and aerobic energy systems. Muscle glycogen and muscle CP stores are significantly lowered, while muscle and blood lactate increase moderately. Despite low external distance, physiological strain was high, likely due to the high number of intense brief efforts. The moderate-to-high internal load is also evidenced by the high heart rate loading with periods with near maximal intensity, although inter-individual differences in aerobic strain were major. Together, these findings support the utility of SSG may be an effective training modality for female players, capable of eliciting sport-specific physiological adaptations. The results also suggest that SSG impose comparable internal load across sexes, reinforcing its role in mixed-sex training and highlighting the need for further investigation into sex-specific training adaptations. It should, however, be noted that due to methodological considerations in relation to not controlling for nutrition and hormonal status, generalization of the results should be done with care.

## Data Availability

The datasets generated and analyzed during the current study contain sensitive personal health information and are therefore not publicly available due to ethical and privacy restrictions. However, anonymized data may be made available from the corresponding author upon reasonable request.
